# Three-Dimensional Incoherent Imaging Using Spiral Rotating Point Spread Functions Created by Double-Helix Beams [Invited]

**DOI:** 10.1186/s11671-022-03676-6

**Published:** 2022-03-24

**Authors:** Vijayakumar Anand, Svetlana Khonina, Ravi Kumar, Nitin Dubey, Andra Naresh Kumar Reddy, Joseph Rosen, Saulius Juodkazis

**Affiliations:** 1 Optical Sciences Center and ARC Training Centre in Surface Engineering for Advanced Materials (SEAM), School of Science, Computing and Engineering Technologies, Swinburne University of Technology, Hawthorn, Melbourne, VIC 3122 Australia; 2 Institute of Physics, University of Tartu, 50411 Tartu, Estonia; 3 Samara National Research University, Samara, Russia 443086; 4 Image Processing Systems Institute—Branch of the Federal Scientific Research Centre, “Crystallography and Photonics” of Russian Academy of Sciences, Samara, Russia 443001; 5 School of Electrical and Computer Engineering, Ben-Gurion University of the Negev, P.O. Box 653, 8410501 Beer-Sheva, Israel; 6 Hee Photonic Labs, Riga, LV-1002 Latvia; 7 Department of Physics of Complex Systems, Weizmann Institute of Science, 7610001 Rehovot, Israel; 8 Tokyo Tech World Research Hub Initiative (WRHI), School of Materials and Chemical Technology, Tokyo Institute of Technology, 2-12-1, Ookayama, Meguro-ku, Tokyo, 152-8550 Japan

**Keywords:** Orbital angular momentum, Incoherent holography, Diffractive optics, Imaging, Microscopy

## Abstract

In recent years, there has been a significant transformation in the field of incoherent imaging with new possibilities of compressing three-dimensional (3D) information into a two-dimensional intensity distribution without two-beam interference (TBI). Most incoherent 3D imagers without TBI are based on scattering by a random phase mask exhibiting sharp autocorrelation and low cross-correlation along the depth axis. Consequently, during reconstruction, high lateral and axial resolutions are obtained. Scattering based-Imaging requires a wasteful photon budget and is therefore precluded in many power-sensitive applications. This study develops a proof-of-concept 3D incoherent imaging method using a rotating point spread function termed 3D Incoherent Imaging with Spiral Beams (3DI^2^SB). The rotation speed of the point spread function (PSF) with displacement and the orbital angular momentum has been theoretically analyzed. The imaging characteristics of 3DI^2^SB were compared with a direct imaging system using a diffractive lens, and the proposed system exhibited a higher focal depth than the direct imaging system. Different computational reconstruction methods such as the Lucy–Richardson algorithm (LRA), non-linear reconstruction (NLR), and the Lucy–Richardson–Rosen algorithm (LRRA) were compared. While LRRA performed better than both LRA and NLR for an ideal case, NLR performed better than both under real experimental conditions. Both single plane imaging, as well as synthetic 3D imaging, were demonstrated. We believe that the proposed approach might cause a paradigm shift in the current state-of-the-art incoherent imaging, fluorescence microscopy, and astronomical imaging.

**Supplementary Information:**

The online version contains supplementary material available at 10.1186/s11671-022-03676-6.

## Introduction

Incoherent holography technologies have an interesting history [1]. In the beginning, incoherent holography systems had quite complicated architectures due to spatial and temporal incoherence. Common path interferometers [2], rotational shear interferometers [3], and conoscopic holography [4] were some of the previously widely used architectures. Even though incoherent holography is capable of broad applicability, the above bulky, complicated configurations hindered realizing its full potential. With the development of active devices, the implementation of incoherent holography became relatively simpler. One well-known compact incoherent holography method was the Fresnel incoherent correlation holography (FINCH) which supported two optical channels in a single physical space and independently modulated one of the channels allowing easy application to fluorescence microscopy [5]. Later, FINCH was found to have a capability to achieve super-resolution by breaking the Lagrange invariant condition, which motivated further research and development in the area of incoherent holography [6]. Today FINCH has evolved into an advanced technique investigated by many prominent researchers across the globe. [7]

While FINCH followed a natural path of evolution, retaining its roots from classical holography, another branch of today’s incoherent holography called coded aperture imaging (CAI) has been parallelly developed based on computational optical methods. The CAI methods have been indirect imaging approaches developed to perform imaging with X-rays and Gamma rays, where manufacturing lenses for these wavelengths is challenging [8, 9]. In this direction, uniformly redundant array (URA) [10], modified URA [11], spectral imaging methods [12] were developed consisting of special patterns for the aperture masks. While the above are useful techniques, a pure phase aperture has many interesting features, such as the average speckle size equal to the diffraction-limited spot size [13, 14]. By recording the speckle intensity patterns for an object and a point object followed by a cross-correlation between the two intensity distributions, the image of the object can be reconstructed closer to the resolution limit of conventional imaging.

But unlike FINCH, CAI methods were initially developed to perform 2D imaging. Another main difference between FINCH and CAI was that FINCH is intense on optical experiments and less intense in computational, but CAI was the opposite. In 2016, a holography method called coded aperture correlation holography (COACH) was developed, which blurred the above distinct differences between FINCH and CAI [15]. COACH was intense in both optics as well as computational parts which connected FINCH and CAI. COACH was able to encode not only 3D spatial but also spectral information effectively [16]. As the 3D imaging capabilities were found to be unaffected even without two-beam interference conditions, COACH dropped the two-beam interference requirement and evolved into Interferenceless COACH (I-COACH) [17]. Later, many such techniques based on I-COACH were reported, such as diffuserCam [18] and scatter plate microscope [19] with different computational reconstruction mechanisms.

Even though the scattering mask-based CAI methods can encode spatial and spectral information in a single shot, the photon budget requirement is enormous compared to conventional lens-based imagers [20]. This problem prevents the implementation of such CAI methods to power-sensitive applications such as astronomical imaging and fluorescence microscopy. A modified approach was developed to overcome this problem; Instead of scattering light uniformly, the light was collected and focused into a random array of points in the sensor plane [21]. In this way, the efficiency of imaging was improved. However, the method could not be applied directly for multiplane imaging without multiplexing masks for the required planes.

In one of the recent attempts of implementing CAI methods in the infrared beamline of the Australian Synchrotron, a better understanding of imaging concepts was obtained [22]. The requirement of a high photon budget and the beam characteristics of Cassegrain objective lenses (COL) resulted in a new imaging path. The light diffracted from COL had four sharp intensity peaks over a large depth, and so the autocorrelation function was sharp along the depth axis while the cross-correlation was lower as the spacing between the spots increased with distance. This is exactly the condition needed for performing indirect 3D imaging. This observation resulted in coining a new optical field condition for 3D imaging with CAI—sharp autocorrelation and low cross-correlation along depth (SALCAD) field. Therefore, the scattered light is random SALCAD field, while the case with COL is a deterministic SALCAD field. For imaging 3D information using the SALCAD fields generated by COL, a special reconstruction algorithm called Lucy–Richardson–Rosen algorithm (LRRA) [23] was developed by combining the non-linear reconstruction method (NLR) developed recently [24] and the well-known Lucy–Richardson algorithm (LRA) [25, 26].

The above study opened the possibility of implementing deterministic SALCAD fields with energy concentrated in a small area similar to conventional imaging but suitable for 3D imaging. In this direction, beams carrying orbital angular momentum (OAM) exhibit interesting characteristics, including SALCAD property. An interesting family of beams is the OAM beams with rotating intensity distributions along the depth axis [27–29]. There have been some reports on implementing rotating point spread functions to improve the localization and sensing along the depth axis [30–34]. Most of the studies [30–32], employed a double helix pattern to improve 3D localization. In conventional fluorescence imaging, when the fluorescent particles lie beyond the depth of field of the imaging system, they appear blurred with a larger size resulting in loss of resolution. By converting every point image into a double helix pattern, the depth of field is increased, and the location of the particle can be identified by the rotation angle of the two spots. This approach improved the 3D localization of fluorescent particles. In 2017, 3D imaging was demonstrated with a double helix pattern using the widely used deconvolution algorithms, namely Weiner filter and the Lucy–Richardson algorithm [33]. But the method was demonstrated only for a short axial distance. In this study, we have investigated the characteristics of 3D Incoherent Imaging using Spiral Beam (3DI^2^SB) and have demonstrated 3D imaging. Two recently developed reconstruction algorithms, namely NLR [24] and LRRA [23] have been applied to expand the limits of 3D imaging [25, 26]. The manuscript consists of five sections. In the second section, the design of the phase mask, theoretical analysis of beam propagation to understand the rate of rotation, and OAM estimation were described. In the third section, simulation results of imaging are presented. The experimental results are presented in the fourth section. The conclusion and future perspectives of this research work are discussed in the final section.

## Methods

**Fig. 1:**
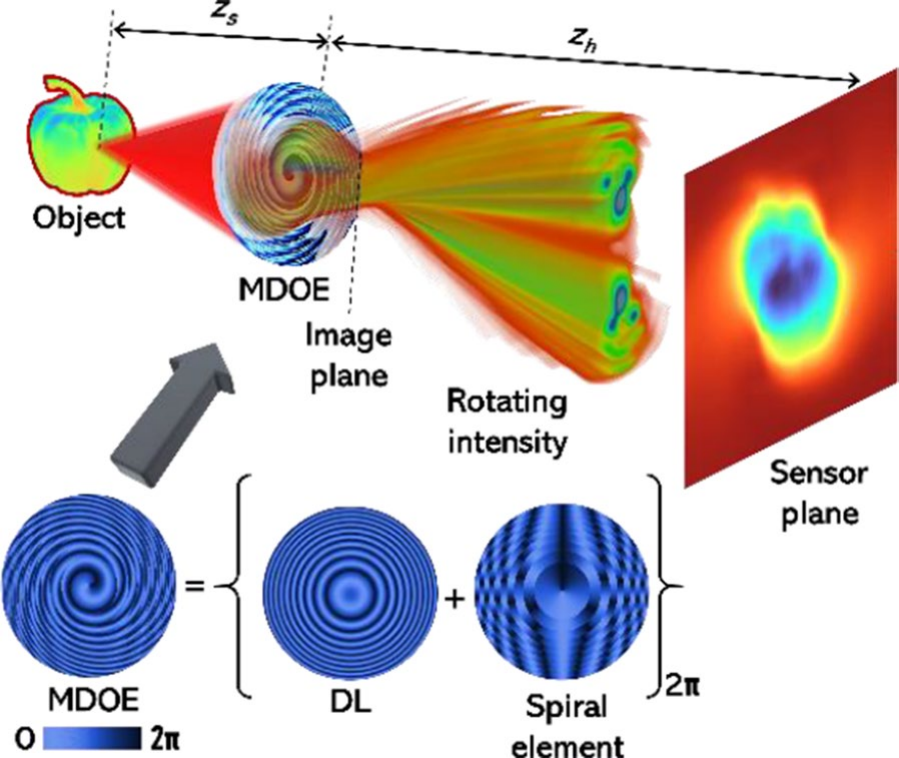
Optical configuration of 3DI^2^SB and generation of phase distribution of the MDOE from a lens and spiral element

The optical configuration of 3DI^2^SB is shown in Fig. 1. Basically, this configuration includes the observed object, a multifunctional diffractive optical element (MDOE) and the image sensor. This section consists of three sections: design and analysis of MDOE, estimation of OAM, and imaging methodology.

### Design and Analysis of MDOE

The MDOE that can generate a rotating intensity distribution has been adapted from Ref. [31]. This study uses spatially incoherent and temporally coherent illumination, so the design and calculation have been carried out for a single wavelength. The MDOE is formed by the modulo-2*π* phase addition of the phase distributions of a diffractive lens (DL) and a spiral element. The topological charge distribution of the spiral element follows an arithmetic progression dependent upon the zone number of annular zones with equal areas starting from the center. Let us consider the MDOE in the following form:

1 $$g(r,\varphi ) = L(r)S(r,\varphi ),$$

where $L(r) = \exp ( - i\pi r^{2} /(\lambda f))$ is the DL in the MDOE and $S(r,\varphi )$ is the spiral element in the following form:

2 $$S(r,\varphi ) = \sum\limits_{n = 1}^{N} {R_{n} (r)\exp \left( {i(2n - 1)\varphi } \right)} ,$$

3 $${\text{where}}\quad R_{n} (r) = \left\{ {\begin{array}{*{20}c} {1,} & {r_{n - 1} < r \le r_{n} ,} \\ {0,} & {{\text{else}}.} \\ \end{array} } \right.\quad r_{n} = \sqrt {2n\lambda f} ,$$

where *r* is the radial coordinate and $\varphi$ is the azimuthal coordinate, *f* is the focal length of the DL given as $1/f = 1/z_{s} + 1/z_{h}$ assuming paraxial approximation [35], *λ* is the wavelength and *n* is the order of circular zones, *z*_*s*_ and *z*_*h*_ are the object distance and image distances, respectively. For a point object with unit amplitude located along the optical axis at *z* = *z*_*s*_ + Δ from the MDOE, where Δ is the defocus distance in the object plane, the complex amplitude after the MDOE using Kirchhoff-Fresnel integral is given as

4 $$E(\rho ,\theta ,z) = - \frac{i}{{\lambda z_{h} }}\int\limits_{0}^{2\pi } {\int\limits_{0}^{R} {g(r,\varphi )\exp \left( {i\frac{{\pi r^{2} }}{\lambda z}} \right)\exp \left\{ {\frac{i\pi }{{\lambda z_{h} }}\left[ {\rho^{2} + r^{2} - 2\rho r\cos (\varphi - \theta )} \right]} \right\}r{\text{d}}r{\text{d}}\varphi } } ,$$

where *R* is the radius of the beam at the MDOE plane.

Considering the focusing part of the MDOE (1), we can rewrite expression (4) as follows:

5 $$E(\rho ,\theta ,z) = - \frac{i}{{\lambda z_{h} }}\exp \left( {\frac{{i\pi \rho^{2} }}{{\lambda z_{h} }}} \right)\int\limits_{0}^{2\pi } {\int\limits_{0}^{R} {\exp \left[ {\frac{{i\pi r^{2} }}{\lambda }\left( {\frac{1}{z} - \frac{1}{{z_{s} }}} \right)} \right]S\left( {r,\varphi } \right)\exp \left[ { - \frac{i2\pi }{{\lambda z_{h} }}\rho r\cos (\varphi - \theta )} \right]r{\text{d}}r{\text{d}}\varphi } } .$$

Obviously, for *z* = *z*_*s*_ we get a focused field, which is the Fourier transform of the spiral element $S(r,\varphi )$, and at *z* ≠ *z*_*s*_ we get a defocused field.

When we substitute (2)–(3) into (5) we obtain:

6 $$\begin{aligned} E(\rho ,\theta ,z) & = - \frac{i}{{\lambda z_{h} }}\exp \left( {\frac{{i\pi \rho^{2} }}{{\lambda z_{h} }}} \right) \\ & \quad \times \sum\limits_{n = 1}^{N} {\int\limits_{0}^{2\pi } {\int\limits_{{r_{n - 1} }}^{{r_{n} }} {\exp \left[ {\frac{{i\pi r^{2} }}{\lambda }\left( {\frac{1}{z} - \frac{1}{{z_{s} }}} \right)} \right]\exp \left( {i\left( {2n - 1} \right)\varphi } \right)\exp \left[ { - \frac{i2\pi }{{\lambda z_{h} }}\rho r\cos (\varphi - \theta )} \right]r{\text{d}}r{\text{d}}\varphi } } } . \\ \end{aligned}$$

For each ring of the spiral element $S\left( {r,\varphi } \right)$, the integration by d$\varphi$ can be performed analytically:

7 $$E(\rho ,\theta ,z) = - \frac{i2\pi }{{\lambda z_{h} }}\exp \left( {\frac{{i\pi \rho^{2} }}{{\lambda z_{h} }}} \right)\sum\limits_{n = 1}^{N} {\left( { - i} \right)^{2n - 1} \exp \left( {i\left( {2n - 1} \right)\theta } \right)\int\limits_{{r_{n - 1} }}^{{r_{n} }} {\exp \left[ {\frac{{i\pi r^{2} }}{\lambda }\left( {\frac{1}{z} - \frac{1}{{z_{s} }}} \right)} \right]J_{2n - 1} \left( {\frac{2\pi }{{\lambda z_{h} }}\rho r} \right)r{\text{d}}r} } .$$

If the rings are sufficiently narrow, then instead of (7) we can approximately write:

8 $$E(\rho ,\theta ,z) \approx \frac{2\pi }{{\lambda z_{h} }}\exp \left( {\frac{{i\pi \rho^{2} }}{{\lambda z_{h} }}} \right)\sum\limits_{n = 1}^{N} {r_{n} \exp \left[ {i\left( {2n - 1} \right)\left( {\theta - \frac{\pi }{2}} \right)} \right]\exp \left[ {\frac{{i\pi r_{n}^{2} \left( {z_{s} - z} \right)}}{{\lambda zz_{s} }}} \right]J_{2n - 1} \left( {\frac{2\pi }{{\lambda z_{h} }}\rho r_{n} } \right)} .$$

Let us rewrite expression (8) in a form convenient for analysis:

9 $$E(\rho ,\theta ,z) \approx A\sum\limits_{n = 1}^{N} {C_{n} \left( {\theta ,z} \right)r_{n} J_{2n - 1} \left( {\frac{2\pi }{{\lambda z_{h} }}\rho r_{n} } \right)} ,$$

where $A = \frac{2\pi }{{\lambda z_{h} }}\exp \left( {\frac{{i\pi \rho^{2} }}{{\lambda z_{h} }}} \right)$, $C_{n} \left( {\theta ,z} \right) = \exp \left[ {i\left( {2n - 1} \right)\left( {\theta - \frac{\pi }{2}} \right)} \right]\exp \left[ {\frac{{i\pi r_{n}^{2} \left( {z_{s} - z} \right)}}{{\lambda zz_{s} }}} \right]$, $z_{s} - z = - \Delta$. Assuming a special 2*f* condition, *z*_*s*_ = *z*_*h*_, $f - z = - \frac{{z_{s} }}{2} - \Delta$. Expression (9) is a superposition of Bessel beams. The behavior of such beams during propagation was studied in various works [27, 29, 32, 36], where it was shown that the rotation of the intensity of the beam (9) is determined by the interference terms and the phase difference:

10 $$\cos \left\{ {2\left( {n_{2} - n_{1} } \right)\left( {\theta - \frac{\pi }{2}} \right) - \frac{{\pi \Delta \left( {r_{{n_{2} }}^{2} - r_{{n_{1} }}^{2} } \right)}}{{2\lambda zz_{s} }}} \right\}.$$

Substituting the expressions for the radii of the rings $r_{n} = \sqrt {2n\lambda f}$, instead of (10) we obtain:

11 $$\cos \left\{ {2\left( {n_{2} - n_{1} } \right)\left( {\theta - \frac{\pi }{2}} \right) - \frac{{\pi f\Delta \left( {n_{2} - n_{1} } \right)}}{{\left( {z_{s} + \Delta } \right)z_{s} }}} \right\}.$$

Then the rotation speed of the beam intensity depending on the distance shift from object Δ will be (omitting the smaller term):

12 $$\frac{{{\text{d}}\theta }}{{{\text{d}}\Delta }} \approx \frac{\pi f}{{2\left( {z_{s} + \Delta } \right)z_{s} }} .$$

Note that displacement Δ can be positive or negative, which means rotation in different directions. Moreover, for large values Δ, the rotation speed will differ depending on the sign due to the nonlinearity of the expression (12). At small displacements, the rotation speed will be linear:

13 $$\frac{{{\text{d}}\theta }}{{{\text{d}}\Delta }} \approx \frac{\pi f}{{2z_{s}^{2} }} .$$

### Calculation of the Orbital Angular Momentum (OAM) of the Field Generated by the Spiral Element

The normalized OAM of an arbitrary scalar field is defined as follows [36]:

14 $$j_{z} = \frac{{J_{z} }}{W} = \left\{ {{\text{Im}} \int\limits_{0}^{\infty } {\int\limits_{0}^{2\pi } {\left[ {E^{*} (r,\varphi ) \cdot \frac{\partial E(r,\varphi )}{{\partial \varphi }}} \right]r{\text{d}}r} {\text{d}}\varphi } } \right\}\left\{ {\int\limits_{0}^{\infty } {\int\limits_{0}^{2\pi } {\left[ {E^{*} (r,\varphi ) \cdot E(r,\varphi )} \right]r{\text{d}}r} {\text{d}}\varphi } } \right\}^{ - 1} .$$

Since the OAM is an invariant characteristic of the field and is conserved during propagation and focusing, the quantity (15) can be calculated in any plane, including the initial plane (at *z* = 0):

15 $$j_{z} = \left\{ {{\text{Im}} \int\limits_{0}^{R} {\int\limits_{0}^{2\pi } {\left[ {g^{*} (r,\varphi ) \cdot \frac{\partial g(r,\varphi )}{{\partial \varphi }}} \right]r{\text{d}}r} {\text{d}}\varphi } } \right\}\left\{ {\int\limits_{0}^{R} {\int\limits_{0}^{2\pi } {\left| {g(r,\varphi )} \right|^{2} r{\text{d}}r} {\text{d}}\varphi } } \right\}^{ - 1} .$$

Since MDOE (1) is a purely phase element, the integral in the denominator of expression (15) corresponds to the energy of a bounded plane illuminating beam $\varepsilon_{0} = \pi R^{2}$. Considering that the focusing part of MDOE (1) does not change the value of OAM, the integral in the numerator of expression (15) can be calculated as follows:

16 $$\begin{aligned} \varepsilon & = \int\limits_{0}^{R} {\int\limits_{0}^{2\pi } {\left[ {g^{*} (r,\varphi ) \cdot \frac{\partial g(r,\varphi )}{{\partial \varphi }}} \right]r{\text{d}}r} {\text{d}}\varphi } = \sum\limits_{n = 1}^{N} {\int\limits_{{r_{n - 1} }}^{{r_{n} }} {\left[ {\int\limits_{0}^{2\pi } {S^{*} (r,\varphi ) \cdot \frac{\partial S(r,\varphi )}{{\partial \varphi }}} {\text{d}}\varphi } \right]r{\text{d}}r} } \\ & = i2\pi \sum\limits_{n = 1}^{N} {\left( {2n - 1} \right)\int\limits_{{r_{n - 1} }}^{{r_{n} }} {r{\text{d}}r} } = i\pi \sum\limits_{n = 1}^{N} {\left( {2n - 1} \right)\left( {r_{n}^{2} - r_{n - 1}^{2} } \right)} . \\ \end{aligned}$$

Considering equality $r_{n} = R\sqrt {n/N}$ [27], we obtain the following expression:

17 $$j_{z} = \frac{1}{N}\sum\limits_{n = 1}^{N} {\left( {2n - 1} \right)} = N.$$

Thus, the normalized OAM of the beams generated by the *N*-zone spiral element (2)–(3) is equal to the number of zones *N*.

### Incoherent Imaging

In the previous sections, we described the design of the DOE, theoretical analysis of the special beam, and estimation of OAM. The above studies were carried out for a single point. This analysis for a single point is true for both coherent as well as incoherent imaging systems. However, when moving on from a single point to a complicated 2D object consisting of multiple points, the description of imaging systems with coherent and incoherent sources are completely different except for some specially imposed conditions of limited field of view and sparse distribution of points [37]. The current imaging configuration converts an object point into a rotating intensity distribution with two main intensity peaks that rotate around the optical axis as a double helix. When the object point’s location changes laterally, the intensity distribution shifts, and when the location changes axially, then the intensity distribution rotates either clockwise or anticlockwise depending upon the direction of the axial shift. The point spread function of the incoherent imaging system is given as $I_{{{\text{PSF}}}} = \left| {E(\rho ,\theta ,z)} \right|^{2}$. In this study, we are interested in creating an incoherent imaging system that is linear in intensity, and so for a 2D object *O*, the intensity distribution is given as $I_{O} = I_{{{\text{PSF}}}} \otimes O$, where ‘⊗’ is the 2D convolutional operator. The image reconstruction is $I_{R} = I_{O} * I_{{{\text{PSF}}}}$, where ‘*’ is the 2D correlation operator. Rewriting the above equation of *I*_*R*_ by substituting the composition of *I*_*O*_, gives; $I_{R} = O \otimes I_{{{\text{PSF}}}} * I_{{{\text{PSF}}}}$, where $I_{{{\text{PSF}}}} * I_{{{\text{PSF}}}}$ is the autocorrelation function that reconstructs the image function. The width of the autocorrelation function is twice that of the diffraction-limited spot size, which is 1.22*λz*_*s*_/*D* in the object plane, assuming a regular correlation, also called matched filter, is selected [38]. Alternative a correlation method with phase-only filter [38] has a relatively sharper autocorrelation function. Recently, the NLR method sharpened the autocorrelation function with a width equal to the diffraction-limited spot size [24]. A spherical lens can create a sharp PSF at the sensor if the imaging condition ${1 \mathord{\left/ {\vphantom {1 f}} \right. \kern-\nulldelimiterspace} f} = {1 \mathord{\left/ {\vphantom {1 {z_{1} }}} \right. \kern-\nulldelimiterspace} {z_{1} }} + {1 \mathord{\left/ {\vphantom {1 {z_{2} }}} \right. \kern-\nulldelimiterspace} {z_{2} }}$ is satisfied, where *f*, *z*_1_, and *z*_2_ are the focal length, point-lens distance, and lens-sensor distance, respectively. However, when the imaging condition is violated, the PSF sharpness decreases. In the proposed 3DI^2^SB, the rotating PSF has sharp intensity peaks over a relatively long distance. While the method does not directly create a well-defined image on the sensor, the autocorrelation function is expected to be sharper, facilitating indirect imaging over a long distance without needing a scattering mask.

## Simulation Results

**Fig. 2:**
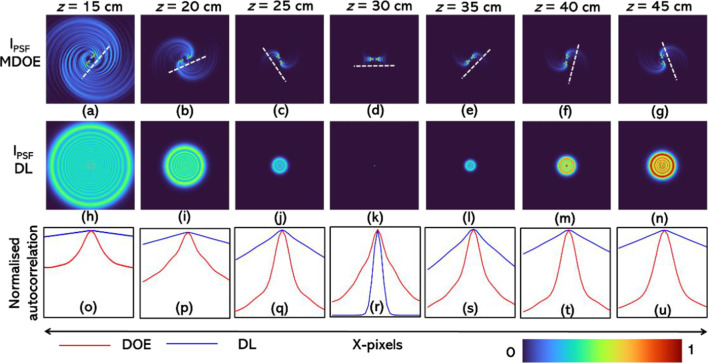
Simulated intensity distributions generated by the MDOE for *z* = **a** 15 cm, **b** 20 cm, **c** 25 cm, **d** 30 cm, **e** 35 cm, **f** 40 cm and **g** 45 cm. Simulated intensity distributions generated by the DL for *z* = **h** 15 cm, **i** 20 cm, **j** 25 cm, **k** 30 cm, **l** 35 cm, **m** 40 cm and **n** 45 cm. The white broken line is attached to (**a**–**g**) to indicate the rotation angle of the spiral beam. The cross-section of the autocorrelation of the intensity distributions (**a**–**n**) are compared in (**o**–**u**), where blue is for DL and red is for MDOE

The simulation studies were carried out for the following specifications of the imaging system. Number of pixels = 500 × 500, *λ* = 600 nm, mesh grid pixel size = 10 μm, *z*_*s*_ = 30 cm, *f* = 15 cm, *z*_*h*_ = 30 cm and *D* = 5 mm. The intensity distribution for *z* = 15 cm to 45 cm in steps of 5 cm for the current system and a reference system consisting of only a DL without a spiral element are shown in Fig. 2a–n, respectively. As seen from Fig. 2a–g, the rotation appears to be faster when the defocus distance in the object plane Δ is smaller, and with a larger increase, the rotation slows down. Secondly, the rotation is faster when the point object is closer to the MDOE. The autocorrelation function is calculated for the case with MDOE and DL, and plotted for the different object locations for MDOE (red) and diffractive lens (blue) in Fig. 2o–u, respectively. It is seen that except for the imaging condition i.e., *z* = 30 cm, for all other cases, the autocorrelation function obtained for the MDOE is sharper with lesser background noise than the case with a DL.

**Fig. 3:**
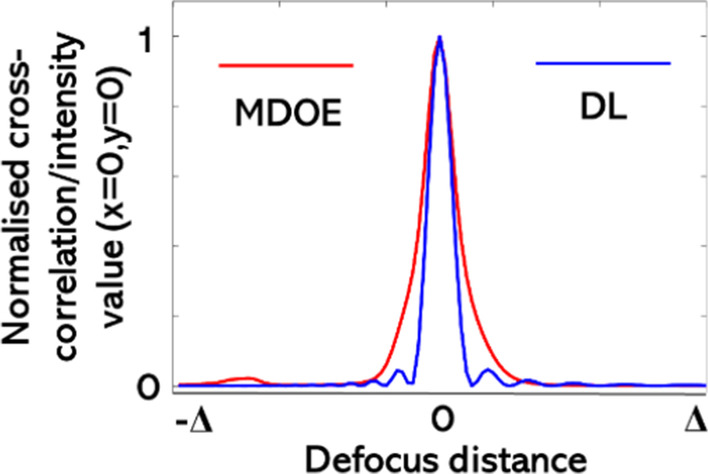
Plot of normalized cross-correlation *C*_*a*_ (*x* = 0, *y* = 0,*z*) (red) and axial intensity of DL along the defocus distance

The axial behavior is studied next. In this study, the simulated intensity distribution at *z* = 30 cm is cross-correlated with matched filter (along the transverse coordinates) with other values from *z* = 15 cm to 45 cm, i.e., $C_{a} = I_{{{\text{PSF}}}} \left( {z = 30\,{\text{cm}}} \right) * I_{{{\text{PSF}}}} \left( z \right)$. The plot of $C_{a} (x = 0,y = 0)$ for the MDOE and the intensity variation for the DL are shown in Fig. 3. The FWHM of the above profile is approximately the axial resolution of the system ~ *λ*/NA^2^, where NA is the numerical aperture. It is seen that the focal depth of the MDOE is longer than that of the DL. In other words, the axial resolution of imaging using a DL is expected to be better than the 3DI^2^SB method. The simulation study reveals two main characteristics of imaging: lateral resolution and axial resolution. The lateral resolution given by the width of the autocorrelation function is better for 3DI^2^SB method than imaging using a DL except for the case when the imaging condition is satisfied. The axial resolution of the 3DI^2^SB method is lower than that of imaging using a DL. The videos of the variation in the intensity distribution for the DL and MDOE are given in Additional file 1: Video S1 and Additional file 2: Videos S2, respectively. The videos of the variation of the intensity distribution of the cross-correlation matrix *C*_*a*_(*x,y,z*) for the MDOE when the distance was varied from *z* = 15 cm to 45 cm are given in Additional file 3: Videos S3.

A test object with the words “Structured light” was selected for the simulation studies. In imaging with a single DL, it is expected that the image resembles the object at the image plane and is blurred anywhere else. In the case of MDOE, the image of the object will probably appear distorted for all distances. It is necessary to reconstruct the image by processing it with the PSF as described in “Incoherent Imaging” section. The processing can be achieved either using cross-correlation between two intensity matrices or by iteratively estimating the maximum likelihood solution as in LRA [25, 26]. In some of the studies involving scattering and with Cassegrain objective lenses, the NLR was found to exhibit a better quality of image reconstruction [22]. In a recent study [23], the LRRA was applied to distorted images recorded with a Cassegrain objective lens and found to perform better than both LRA and NLR. All three reconstruction methods mentioned above have not yet been applied to an exotic intensity distribution such as the rotating PSF.

**Fig. 4:**
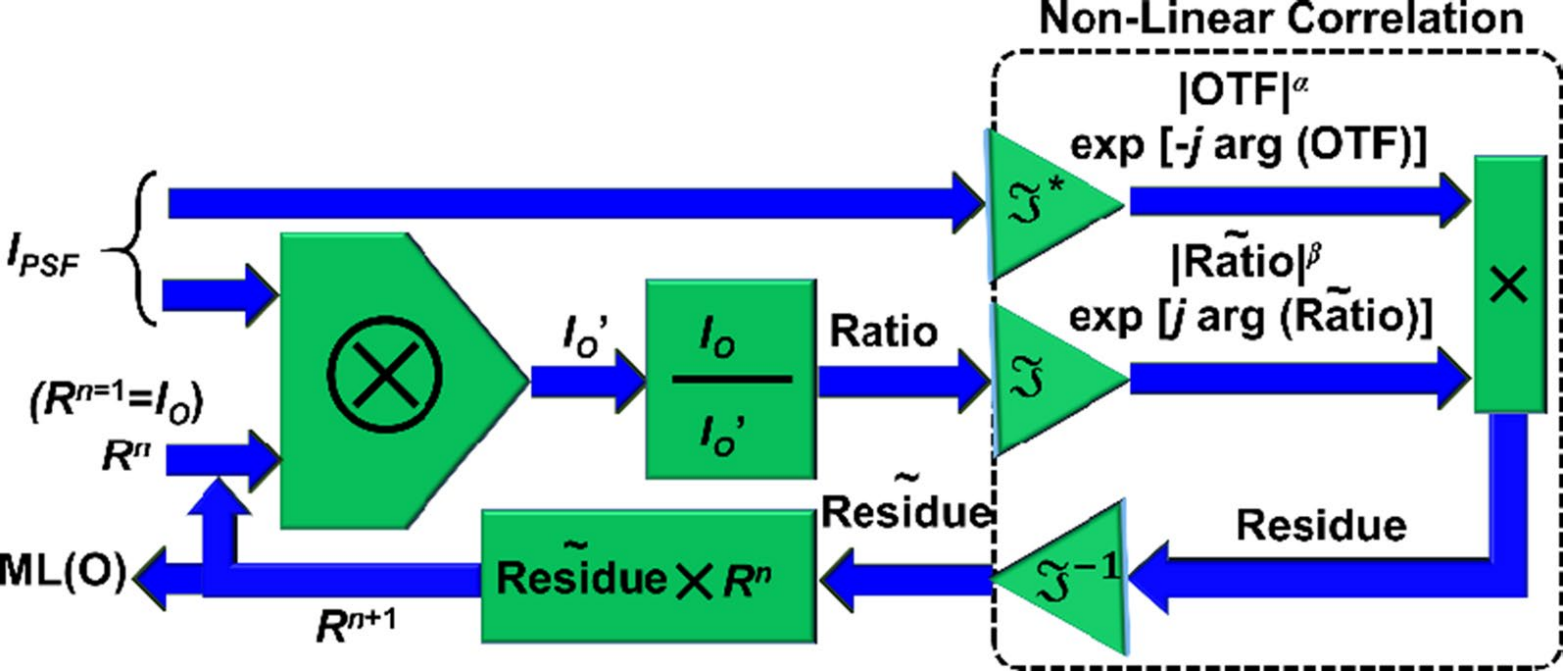
Schematic of the LRRA. When *α* = *β* = 1, LRRA becomes LRA

The NLR is given as $I_{R} = \left| {{\mathcal{F}}^{ - 1} \left\{ {\left| {\tilde{I}_{{{\text{PSF}}}} } \right|^{\alpha } \exp \left[ {i \arg \left( {\tilde{I}_{{{\text{PSF}}}} } \right)} \right]\left| {\tilde{I}_{O} } \right|^{\beta } \exp \left[ { - i \arg \left( {\tilde{I}_{O} } \right)} \right]} \right\}} \right|$, where *α* and *β* are tuned between − 1 and 1, to obtain the minimum entropy given as $S\left( {p,q} \right) = - \sum \sum \phi \left( {m,n} \right)\log \left[ {\phi \left( {m,n} \right)} \right].$ $\phi \left( {m,n} \right) = \left| {C\left( {m,n} \right)} \right|/\sum\nolimits_{M} {\sum\nolimits_{N} {\left| {C\left( {m,n} \right)} \right|} }$, where (*m,n*) are the indexes of the correlation matrix, and *C*(*m,n*) is the cross-correlation distribution. The LRRA is shown in Fig. 4. The LRRA has three parameters namely *α*, *β* and number of iterations. When *α* = 1, *β* = 1, the algorithm is LRA.

**Fig. 5:**
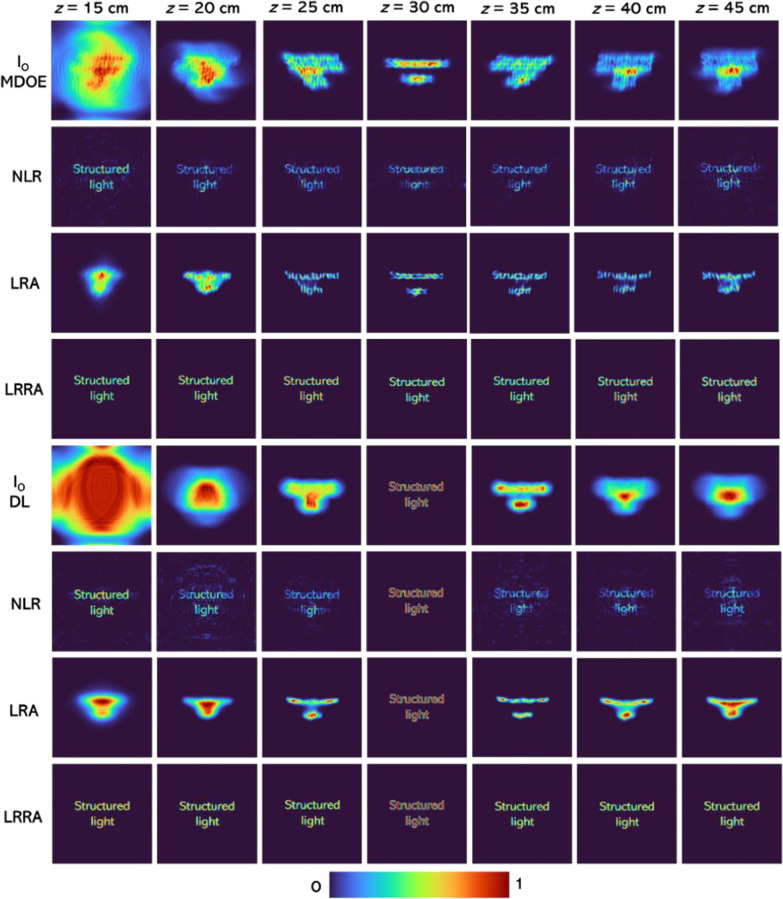
Simulated intensity distributions for the test object for MDOE and DL and reconstruction results from NLR, LRA and LRRA

The intensity distributions for the test object were simulated for both DL and MDOE for distances from *z* = 15 cm to 45 cm, as shown in Fig. 5. The reconstruction results using NLR reconstruction (*α* =  − 0.3, *β* = 0.6), LRA (iterations = 50) and LRRA (*α* = 0, *β* = 0.5 to 0.7, iterations = 6) are shown in Fig. 5. In all these reconstruction methods, each transverse plane is reconstructed with its own unique PSF. The optimal reconstruction (*α* and *β*) for NLR was determined when the entropy of the reconstructed image was minimal. The optimal iteration number for LRA and iteration number, *α* and *β* values of LRRA were obtained when the difference between the reconstructed image and test object was minimal. The number of parameters to be optimized is one (number of iterations), two (*α* and *β*), and three (all the above) for LRA, NLR, and LRRA, respectively. However, once the values are optimized, for one case, the values remained almost the same when the experimental conditions were not changed. It must be noted that LRRA is obtained by applying NLR to LRA, so LRRA does not require any special workflow but just an additional parameter to optimize. The library of PSFs is computed by displaying a point object in the system input. Knowing its number in the library list for the PSF yielding the best in-focus image enables one to locate the observed object along the *z*-axis.

**Fig. 6:**
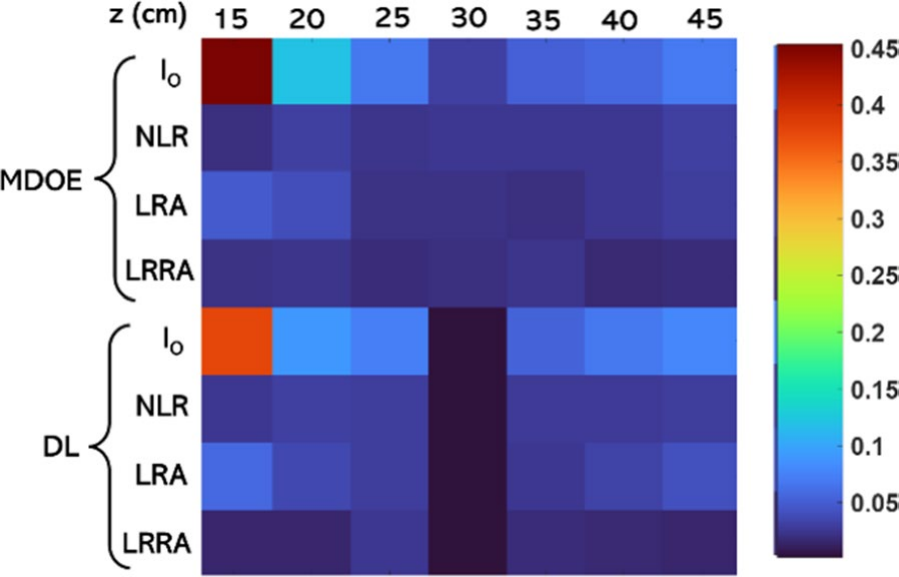
MSE map for the different cases of Fig. 5 with respect to the reference image of the test object obtained by direct imaging using DL

**Fig. 7:**
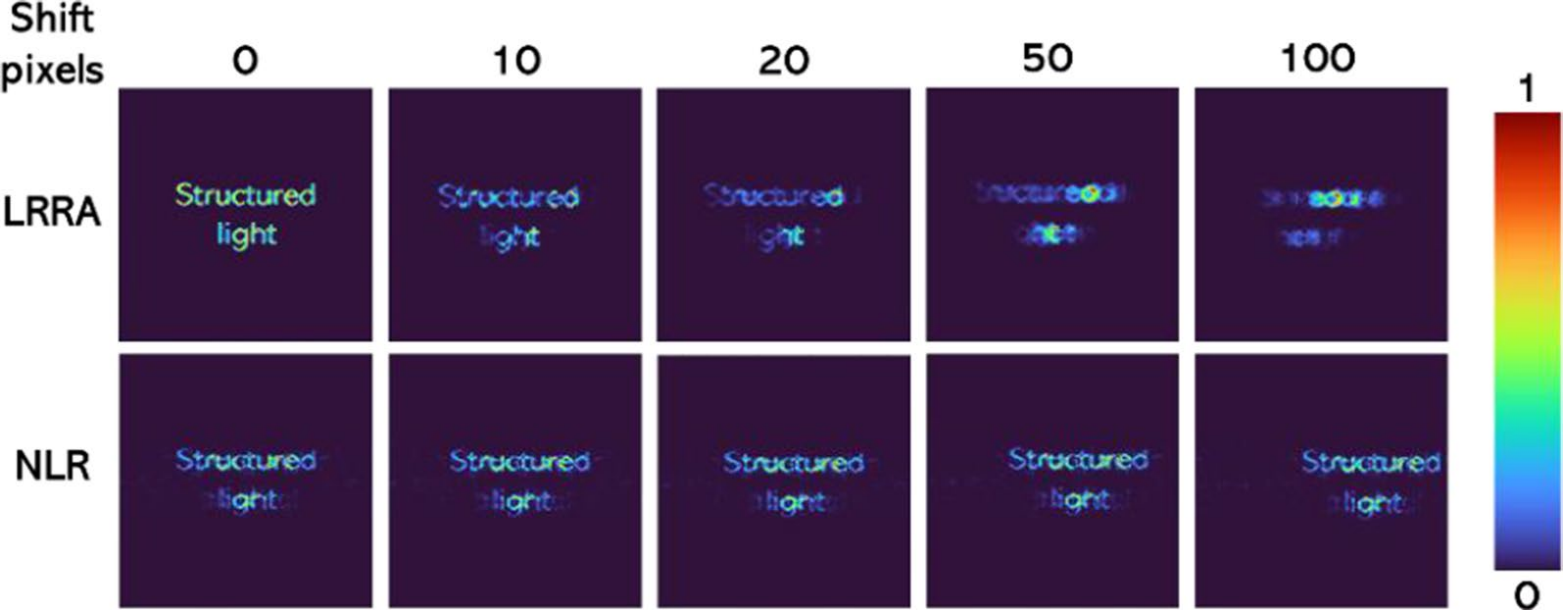
Reconstruction results using LRRA and NLR during a shift error of 0, 10, 20, 50 and 100 pixels

It is seen that NLR always performs better than LRA while LRRA performs better than both. One disadvantage of LRRA is that it involves a certain number of iterations even though significantly lower than LRA, which can prevent the application of LRRA to real-time imaging as NLR. Another observation is that the performance of LRA for MDOE is better than DL. The mean squared error (MSE) was calculated using the direct imaging of the test object using a DL as a reference. The MSE distribution for the different cases is shown in Fig. 6. An important observation of LRRA was that it is highly sensitive to the relative locations of the *I*_PSF_ and *I*_*O*_. The reconstruction methods LRRA and NLR are compared next when there is a relative horizontal shift error of 5, 10, 20, and 50 pixels between *I*_PSF_ and *I*_*O*_. The reconstruction results of NLR and LRRA are shown in Fig. 7. It is seen that when there is a shift error, the performance of LRRA is significantly affected, while in the case of NLR, there is only a shift in the location of the reconstructed information. The previous success of LRRA [23] on the data from the Australian synchrotron can be attributed to the lower number of pixels in the image sensor (64 × 64 pixels), in which the chances of error are lower. However, in most imaging and holography experiments, the scientific camera consists of at least 1 Megapixels. Consequently, LRRA may not be suitable for such cases without additional stabilization approaches to improve the resilience of LRRA in the presence of errors.

## Experiments

**Fig. 8:**
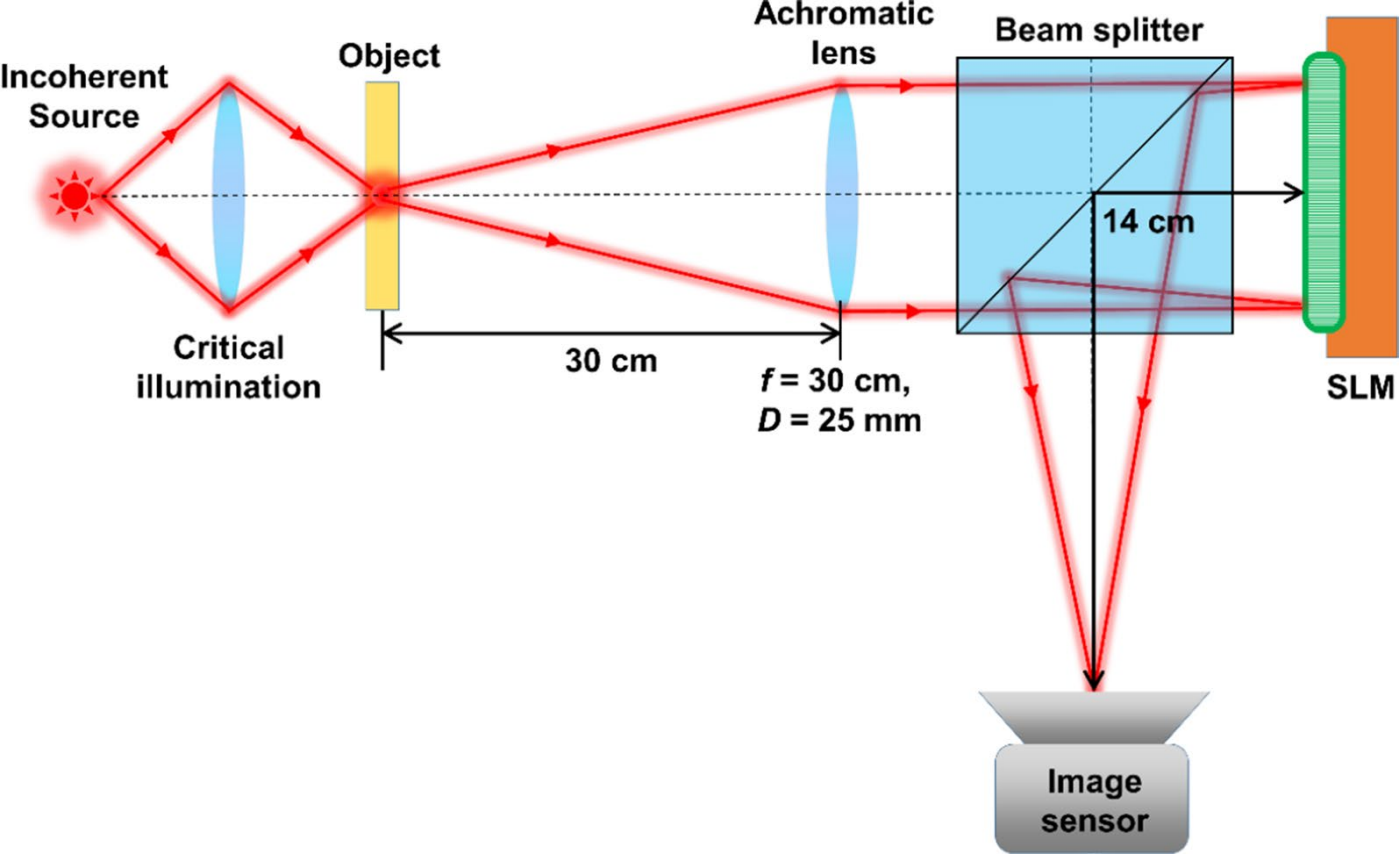
Schematic of the experimental setup. SLM—Spatial light modulator; *f*—focal length and *D*—diameter of the lens

**Fig. 9:**
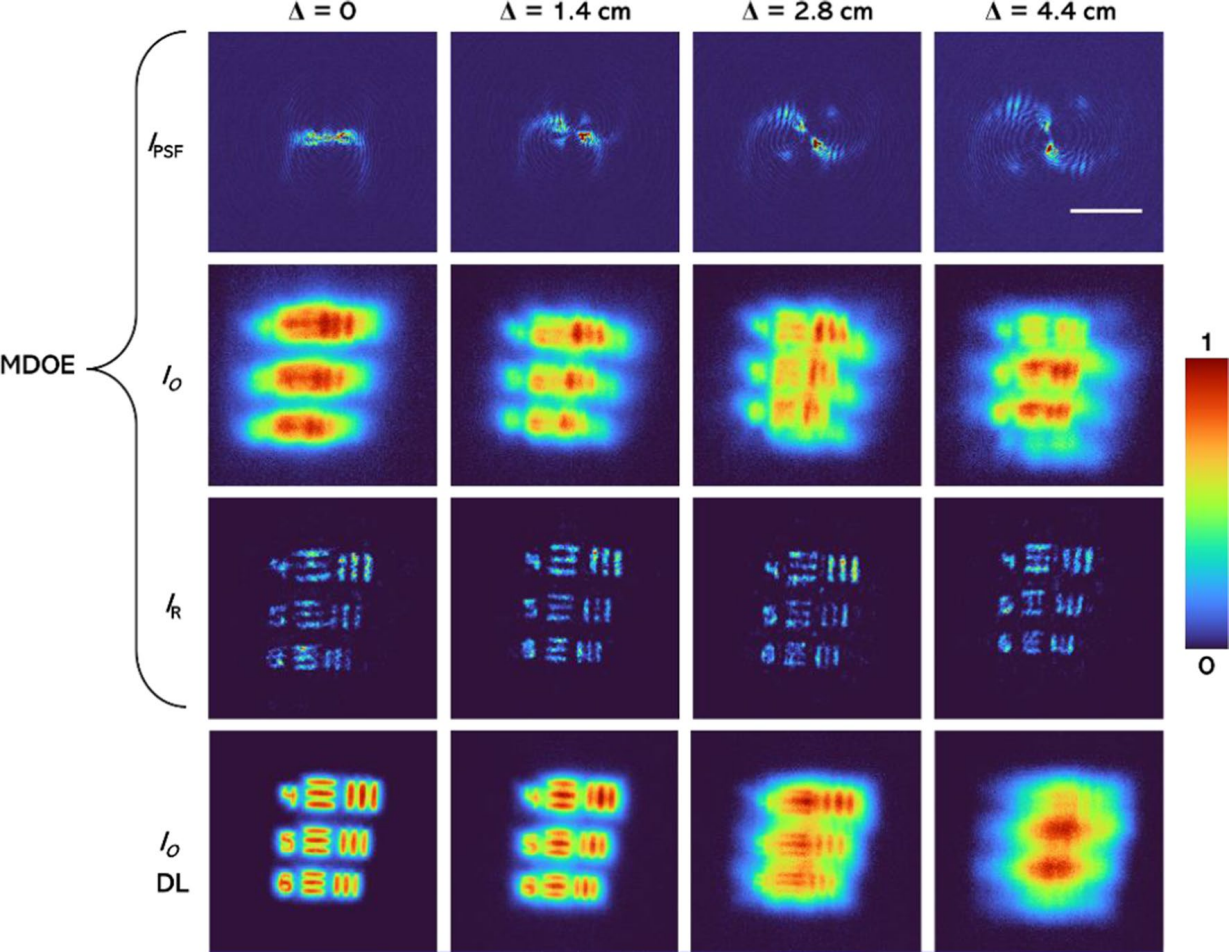
Experimentally recorded *I*_PSF_, *I*_*O*_, and the reconstruction results using NLR with (*α* = 0, *β* = 0.4) and a median filter for the MDOE and reference images obtained for DL. The scale bar is 0.5 mm

The schematic of the experimental setup is shown in Fig. 8. The same optical setup was used for both 2D and 3D experiments. An incoherent source (Thorlabs LED625L, 12 mW, *λ* = 625 nm, ∆*λ* = 15 nm) was used for illuminating the object, elements 4, 5, and 6 (both digits and gratings) of group 5 of negative USAF target were chosen as test object for the experiments. The SLM (Holoeye PLUTO, 1920 × 1080 pixels, 8 µm pixel pitch, phase-only modulation) was used to modulate the light beam by displaying the vortex phase mask shown in Fig. 1, along with the lens function having a focal length of 14 cm. The distance between the SLM and the digital camera (Retiga R6-DCC3260M, pixel size 4.54 μm × 4.54 μm) was 14 cm. A polarizer was used to allow light only along the active axis of the SLM. A pinhole with a size of 15 µm was used to record the *I*_PSF_s. The location of the pinhole was shifted by Δ = 0, 1.4, 2.8, and 4.4 cm and the PSF library was recorded. The USAF object was then mounted at exactly the same locations as the pinhole and the object intensity distributions were recorded. The images of the recorded *I*_PSF_ and *I*_O_ (the system response to an object in the input) for Δ = 0, 1.4, 2.8, and 4.4 cm and the reconstruction results using NLR (*α* = 0, *β* = 0.4) followed by the application of a median filter for MDOE are shown in Fig. 9. The reference images recorded for DL Δ = 0, 1.4, 2.8, and 4.4 cm are also shown in Fig. 9. As seen, the images obtained using MDOE are sharper in comparison to the ones obtained using DL.

**Fig. 10:**
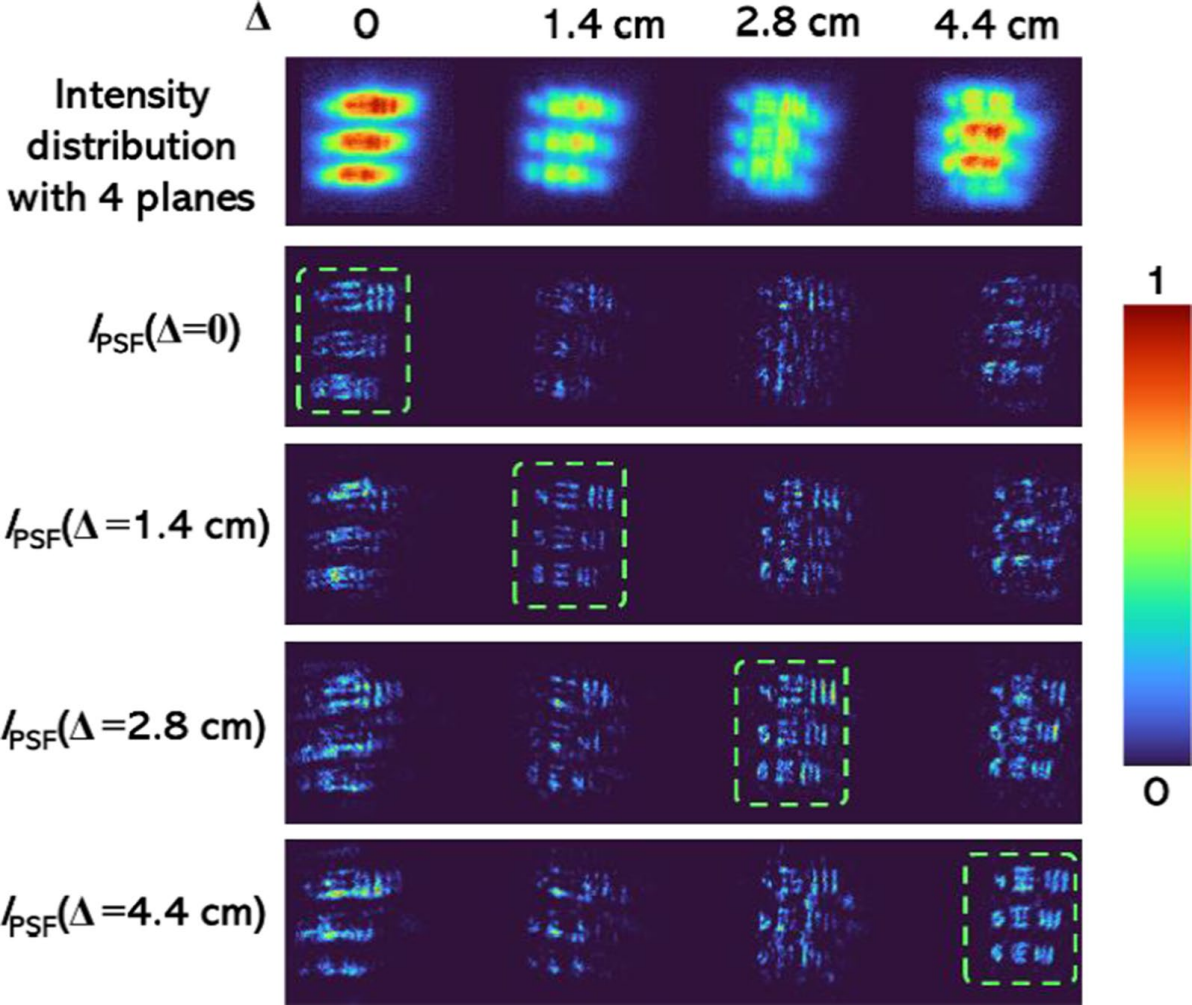
Output images of the system in response to the resolution chart in the input, and reconstruction results using *I*_PSF_ recorded at the respective planes of the objects

For 3D experiments, the fundamental properties of incoherent imaging—the linearity and space invariance in intensity were exploited. It is necessary to understand what a 3D object is. When the object points exist along the *z* direction, it is a 3D object. Some 3D objects have points continuously along *z* while others have points distributed in only a few planes. In our study, we used the 2D data recorded at different axial locations to test the 3D imaging capabilities of the method. The images of the intensity distributions from four separated planes at Δ = 0, 1.4, 2.8, and 4.4 cm are shown in the upper line of Fig. 10. The reconstructed images using the *I*_PSF_ recorded at all four planes are shown in lines 2–5 of Fig. 10. It is seen that only the object information at the planes of the *I*_PSF_ was in focus, while the information from other planes was blurred. The experiment described in Fig. 10 is identical to recording the total intensity distribution simultaneously from multiple planes of an object. In the current experiment, the out-of-focus and the in-focus intensities are computed in the computer instead of appearing on the sensor.

## Discussion and Conclusion

In this study, a new incoherent imaging system, 3DI^2^SB, is investigated. The concept has been proposed as a possible replacement for the existing scattering-based 3D imaging systems [17]. The 3DI^2^SB approach has been studied only for a beam consisting of two intensity peaks that rotate about the optical axis. Apparently, the two peaks exist over a long focal depth enabling a higher signal-to-noise ratio than scattering-based imagers and also a DL. The lateral resolution given by the autocorrelation function was found to be sharper for 3DI^2^SB than the case with a DL. The focal depth of the 3DI^2^SB was found to be slightly higher than that of the imager using a diffractive lens. Three different reconstruction methods, namely LRA, NLR, and LRRA, were compared. LRRA and NLR were found to perform better than LRA. For an ideal case, LRRA performed better than NLR, while for a practical case, NLR performed better than LRRA. The results shown in Fig. 5 show an interesting result. When the autocorrelation function of 3DI^2^SB and regular imaging system with DL were compared, the former was found to be sharper than that of the case with DL. But upon applying NLR and LRRA, the correlation function became shaper for both cases. However, we expect that in practice, with various kinds of noise, the performance of 3DI^2^SB with any reconstruction method will be better because the intensity distribution is more concentrated on a small area along with its propagation than the case of the DL. Further studies are needed in both NLR and LRRA to improve the reconstruction results. We believe that our study will open a pathway for implementing different incoherent exotic beams and structured light for multidimensional and multispectral imaging technologies [20].

## Supplementary Information

**Additional file 1.** The video of the variation of the intensity distribution with distance for the DL.

**Additional file 2.** The video of the variation of the intensity distribution with distance for the MDOE.

**Additional file 3.** The video of the variation of the intensity distribution of the cross-correlation matrix *C*_*a*_ (*x*, *y*, *z*) for the MDOE when the distance was varied from *z* = 15 cm to 45 cm.

## Data Availability

All data generated or analysed during this study are included in this published article.

## Abbreviations

3DI^2^SB: Three-dimensional incoherent imaging using spiral beams
CAI: Coded aperture imaging
COACH: Coded aperture correlation holography
DL: Diffractive lens
FINCH: Fresnel incoherent correlation holography
I-COACH: Interferenceless coded aperture correlation holography
LED: Light emitting diode
LRA: Lucy–Richardson algorithm
LRRA: Lucy–Richardson–Rosen algorithm
MDOE: Multifunctional diffractive optical element
NLR: Non-linear reconstruction
OAM: Orbital angular momentum
PSF: Point spread function
SALCAD: Sharp autocorrelation and low cross-correlation along depth
SLM: Spatial light modulator
TBI: Two beam interference
URA: Uniformly redundant array
MURA: Modified uniformly redundant array
USAF: United States Air Force

## References

[1] Rosen J, Vijayakumar A, Kumar M, Rai MR, Kelner R, Kashter Y, Bulbul A, Mukherjee S (2019). Recent advances in self-interference incoherent digital holography. Adv Opt Photonics.

[2] Peters PJ (1966). Incoherent holograms with a mercury light source. Appl Phys Lett.

[3] Bryngdahl O, Lohmann A (1970). Variable magnification in incoherent holography. Appl Opt.

[4] Sirat G, Psaltis D (1985). Conoscopic holography. Opt Lett.

[5] Rosen J, Brooker G (2007). Digital spatially incoherent Fresnel holography. Opt Lett.

[6] Rosen J, Siegel N, Brooker G (2011). Theoretical and experimental demonstration of resolution beyond the Rayleigh limit by FINCH fluorescence microscopic imaging. Opt Express.

[7] Rosen J (2021). Roadmap on recent progress in finch technology. J Imaging.

[8] Ables JG (1968). Fourier transform photography: a new method for X-ray astronomy. Publ Astron Soc Austral.

[9] Dicke RH (1968). Scatter-hole cameras for X-rays and gamma rays. Astrophys J.

[10] Fenimore EE, Cannon TM (1981). Uniformly redundant arrays: digital reconstruction methods. Appl Opt.

[11] Gottesman SR, Fenimore EE (1989). New family of binary arrays for coded aperture imaging. Appl Opt.

[12] Wagadarikar A, John R, Willett R, Brady D (2008). Single disperser design for coded aperture snapshot spectral imaging. Appl Opt.

[13] Lee K, Park Y (2016). Exploiting the speckle-correlation scattering matrix for a compact reference-free holographic image sensor. Nat Commun.

[14] Dainty JC (2013). Laser speckle and related phenomena.

[15] Vijayakumar A, Kashter Y, Kelner R, Rosen J (2016). Coded aperture correlation holography—a new type of incoherent digital holograms. Opt Express.

[16] Vijayakumar A, Rosen J (2017). Spectrum and space resolved 4D imaging by coded aperture correlation holography (COACH) with diffractive objective lens. Opt Lett.

[17] Vijayakumar A, Rosen J (2017). Interferenceless coded aperture correlation holography—a new technique for recording incoherent digital holograms without two-wave interference. Opt Express.

[18] Antipa N, Kuo G, Heckel R, Mildenhall B, Bostan E, Ng R, Waller L (2018). DiffuserCam: lensless single-exposure 3D imaging. Optica.

[19] Singh AK, Pedrini G, Takeda M, Osten W (2017). Scatter-plate microscope for lensless microscopy with diffraction limited resolution. Sci Rep.

[20] Anand V, Ng SH, Maksimovic J, Linklater D, Katkus T, Ivanova EP, Juodkazis S (2020). Single shot multispectral multidimensional imaging using chaotic waves. Sci Rep.

[21] Rai MR, Rosen J (2019). Noise suppression by controlling the sparsity of the point spread function in interferenceless coded aperture correlation holography (I-COACH). Opt Express.

[22] Anand V, Ng SH, Katkus T, Maksimovic J, Klein AR, Vongsvivut J, Bambery KR, Tobin MJ, Juodkazis S (2021). Exploiting spatio-spectral aberrations for rapid synchrotron infrared imaging. J Synchrotron Radiat.

[23] Anand V, Han M, Maksimovic J, Ng SH, Katkus T, Klein AR, Bambery KR, Tobin MJ, Vongsvivut J, Juodkazis S (2022). Single-shot mid-infrared incoherent holography using Lucy Richardson Rosen algorithm. Opto-Electron Sci.

[24] Rai MR, Vijayakumar A, Rosen J (2018). Non-linear adaptive three-dimensional imaging with interferenceless coded aperture correlation holography (I-COACH). Opt Express.

[25] Richardson WH (1972). Bayesian-based iterative method of image restoration. JoSA.

[26] Lucy LB (1974). An iterative technique for the rectification of observed distributions. Astron J.

[27] Kotlyar VV, Khonina SN, Skidanov RV, Soifer VA (2007). Rotation of laser beams with zero of the orbital angular momentum. Opt Commun.

[28] Schulze C, Roux FS, Dudley A, Rop R, Duparré M, Forbes A (2015). Accelerated rotation with orbital angular momentum modes. Phys Rev A.

[29] Kotlyar VV, Soifer VA, Khonina SN (1997). An algorithm for the generation of laser beams with longitudinal periodicity: rotating images. J Mod Opt.

[30] Pavani SRP, Piestun R (2008). Three dimensional tracking of fluorescent microparticles using a photon-limited double-helix response system. Opt Express.

[31] Prasad S (2013). Rotating point spread function via pupil-phase engineering. Opt Lett.

[32] Baránek M, Bouchal Z (2014) Optimizing the rotating point spread function by SLM aided spiral phase modulation. In: 19th Polish–Slovak–Czech optical conference on wave and quantum aspects of contemporary optics, vol 9441. International Society for Optics and Photonics, p 94410N

[33] Wang Z, Cai Y, Liang Y, Zhou X, Yan S, Dan D, Bianco PR, Lei M, Yao B (2017). Single shot, three-dimensional fluorescence microscopy with a spatially rotating point spread function. Biomed Opt Express.

[34] Wang W, Situ G (2017). Interferometric rotating point spread function. Sci Rep.

[35] Vijayakumar A, Bhattacharya S (2013). Characterization and correction of spherical aberration due to glass substrate in the design and fabrication of Fresnel zone lenses. Appl Opt.

[36] Khonina SN, Kotlyar VV, Soifer VA, Pääkkönen P, Simonen J, Turunen J (2001). An analysis of the angular momentum of a light field in terms of angular harmonics. J Mod Opt.

[37] Hai N, Rosen J (2019). Interferenceless and motionless method for recording digital holograms of coherently illuminated 3D objects by coded aperture correlation holography system. Opt Express.

[38] Horner JL, Gianino PD (1984). Phase-only matched filtering. Appl Opt.

